# Quantitative Characterization of Corneal Collagen Architecture Using Intensity Gradient Modeling and Gaussian PDF Fitting

**DOI:** 10.3390/diagnostics15141738

**Published:** 2025-07-08

**Authors:** Enrique J. Fernandez, Juan M. Bueno

**Affiliations:** Laboratorio de Óptica, Instituto Universitario de Investigación en Óptica y Nanofísica, Campus de Espinardo (Ed. 34), CEIR Campus Mare Nostrum (CMN), Universidad de Murcia, 30100 Murcia, Spain; bueno@um.es

**Keywords:** corneal collagen organization, microscopy, intensity gradient analysis

## Abstract

**Background/Objectives:** The transparency and biomechanical properties of the human cornea are governed by the precise organization of collagen fibers. A novel quantitative technique to analyze corneal collagen organization, based on intensity gradient modeling and probability density function (PDF) fitting, is proposed. **Methods:** Derived from second-harmonic generation (SHG) images, the method calculates image gradients, derives PDFs of gradient orientations, and fits them to Gaussian models. **Results:** Tested across species and temporal healing stages, this approach is an advantageous alternative to traditional methods like Fourier transform and structure tensor analyses, particularly in noisy or low-contrast conditions. **Conclusions:** The technique offers a scalable, robust framework suitable for research, clinical diagnostics, and treatment monitoring.

## 1. Introduction

### 1.1. Corneal Structure

The human cornea is a highly specialized, transparent tissue that accounts for about two-thirds of the eye’s total refractive power and is essential for proper vision. Its transparency, refractive function, and mechanical strength are regulated by the precise arrangement of collagen fibers within the stroma, which makes up nearly 90% of corneal thickness [1,2,3].

The stroma is primarily composed of type I collagen organized into lamellae, where fibers run parallel within each layer and shift orientation across successive layers to maintain optical clarity and biomechanical integrity [4,5]. While both the cornea and the sclera are mainly composed of type I collagen, only the former remains transparent. This is due to the highly ordered arrangement of the corneal collagen, in contrast to the more nonorganized pattern within the sclera [2,6]. In the cornea, collagen fibers are preferentially aligned along the superior–inferior and temporal–nasal axes and arranged into alternating lamellae, with orientation shifts between anterior and posterior regions. At the posterior periphery, collagen fibers adopt a radial configuration critical for maintaining corneal shape and optical properties [2,3,5,7].

Disruptions in this lamellar architecture—whether congenital, degenerative, or trauma-induced—can lead to sight-threatening conditions such as keratoconus, corneal ectasia, scarring, and postsurgical complications [8,9]. A change in the orientation and structure of the collagen matrix, as seen in conditions such as keratoconus, has been proposed to result in corneal biomechanical weakening, which leads to changes in corneal shape and a degradation in its optical properties [6]. Furthermore, a competing theory on keratoconus suggests that alterations in the interweaving of the anterior stroma with the Bowman layer are the primary cause of corneal instability [8]. A common treatment for keratoconus is corneal cross-linking, where a photosensitizer, typically riboflavin, is used in combination with UVA light to stiffen the cornea. Other photosensitizers like Rose Bengal, excited with green light, have been explored for this purpose [9,10]. Studies have shown that mechanical stiffness increases in corneas after cross-linking treatment [10,11,12].

### 1.2. Imaging the Cornea

Advancements in non-linear optical imaging, particularly second-harmonic generation (SHG) and two-photon excited fluorescence (TPEF) microscopy, have enabled the high-resolution, label-free visualization of collagen structures within the cornea in both ex vivo and in vivo settings [4,13,14,15,16,17].

SHG imaging is uniquely suited to visualize collagen due to its sensitivity to non-centrosymmetric molecular arrangements and has been extensively applied in ophthalmology to investigate pathological remodeling, wound healing, and corneal biomechanics [18,19,20,21]. The coherent nature of SHG allows imaging of fibrillar structures with minimal photo-damage and high penetration depth, facilitating repeated, longitudinal assessments [22,23,24]. TPEF complements SHG by visualizing endogenous fluorophores, thus providing additional insights into tissue composition and health [25,26].

### 1.3. Quantitative Analysis of the Corneal Images and Limitations

Despite these powerful imaging capabilities, quantitative analysis of corneal collagen organization from SHG and TPEF images remains a significant technical challenge. A variety of computational methods have been proposed to estimate orientation, alignment, and organization of the corneal collagen fibers. These include Fourier-transform (FT) methods [27,28,29,30,31], structure tensor (ST) analysis [20,32,33], Radon transformation [7,34], gray-level co-occurrence matrix (GLCM)-based texture analysis [19], wavelet transforms [22], and polarization-resolved imaging [15,35,36,37,38]. Each approach aims to provide quantitative descriptors of fiber orientation and distribution, yet each suffers from critical limitations in the context of biological variability and imaging noise.

FT-based techniques are among the most widely used for collagen analysis due to their capacity to decompose spatial patterns into frequency components, thus enabling the estimation of predominant fiber orientations and anisotropy [29,39]. However, FT methods are intrinsically global, lacking the spatial specificity required to detect localized disruptions in collagen alignment typical of early-stage pathologies. Additionally, FT analyses are highly sensitive to noise, especially when applied to biological images with low signal-to-noise ratios, as seen in advanced keratoconus or scarred tissue [40].

To mitigate these limitations, ST analysis has been employed as a local method for mapping orientation distributions based on image gradients, facilitating the detection of local anisotropy [32,41,42]. Nevertheless, ST is also susceptible to artifacts in low-contrast regions and requires careful parameter tuning to balance sensitivity and specificity.

GLCM-based texture analysis has emerged as a complementary strategy to capture second-order statistical patterns in collagen images, offering metrics such as contrast, homogeneity, and correlation [43,44,45]. While GLCM can distinguish between organized and disorganized tissue regions, its indirect relation to fiber orientation and reliance on empirically chosen spatial parameters limit its applicability to quantitative collagen analysis [46].

Similarly, approaches employing wavelet-based decompositions [47] and Radon transforms [7,48,49] offer multi-scale analysis, but they demand extensive preprocessing and are computationally intensive, hindering their real-time or high-throughput application. Methods requiring polarization-resolved SHG for anisotropy quantification, though powerful, are unsuitable for clinical applications due to prolonged acquisition times and sensitivity to motion artifacts. Moreover, many of these approaches focus solely on measuring orientation distributions without assessing fiber continuity and network integrity, crucial elements of tissue mechanics [50,51].

### 1.4. Purpose and Scope of the Work

The unique lamellar architecture of the cornea, composed of hundreds of stacked, quasi-parallel collagen sheets with varying orientations, presents distinct analytical challenges. In certain pathological conditions such as keratoconus, alterations occur not only in the preferred orientation of fibers but also in the continuity, branching, and intersection of lamellae phenomena poorly captured by global orientation metrics.

Despite advancements in imaging, the lack of robust and localized analysis tools limits the quantification of collagen fiber alignment and orientation. Our work addresses this gap by developing a gradient-based statistical method tailored for SHG image analysis. The method is relatively simple to implement, detecting subtle pattern features, providing quantitative classification and understanding of biomedical images.

## 2. Materials and Methods

### 2.1. SHG Microscope and Image Acquisition

A modified inverted microscope (Nikon TE2000-U, Nikon Corporation, Tokyo, Japan) was used to acquire SHG images of the corneal stroma [52]. The microscope was coupled to an 800 nm mode-locked Ti:Sapphire laser (Mira 900, Coherent, St. Clara, CA, USA) that delivered ~150 fs pulses at a 76 MHz repetition rate. The laser beam was directed to the microscope through an optical relay system consisting of a neutral density filter wheel to adjust laser power, a beam expander to triple the beam diameter, and a pair of non-resonant galvanometric mirrors (VM1000, General Scanning Inc - GSI Lumonics, Billerica, MA, USA) for XY scanning. Two scanning resolutions were used: 256 and 512 pixels per line in both XY dimensions. Two telescopes optically conjugated the galvanometric mirrors with the microscope objective’s entrance pupil. The laser power at the sample plane ranged from 30 to 80 mW, depending on the experiment. Inside the microscope, a dichroic mirror reflected the IR excitation beam to the objective (Nikon ELDW Series; 20×, 0.5 NA, Nikon Corporation) and transmitted shorter-wavelength emission signals. The objective focused the laser on the sample and enabled spot scanning. Samples were positioned on a motorized stage for precise XY positioning, and a step-motor controlled *Z*-axis location. Nonlinear signals emitted by the sample were collected back through the same objective and detected by a photomultiplier tube (PMT, R7205-01, Hamamatsu, Bridgewater, NJ, USA), coupled with a photon-counting unit (C6465, Hamamatsu). A bandpass filter (FB400-10, Thorlabs, Newton, NJ, USA) isolated the SHG signal. The PMT output was digitized by a data acquisition card (PCI-6259, National Instruments, Austin, TX, USA) and processed on a PC. Image acquisition and scanner control were managed using custom LabVIEW software (LabVIEW 2009, National Instruments).

### 2.2. Corneal Samples

Healthy corneas from different animal models, including humans, were used in the first part of this study. Porcine and bovine ocular globes were obtained from a local slaughterhouse. Rabit and avian corneas were provided by the Department of Cellular Biology and Histology of the Universidad de Valladolid, Spain. Human corneas from donors not suitable for transplantation were kindly provided by the “Servicio de Oftalmología,” Hospital Universitario Virgen de la Arrixaca, Murcia, Spain.

The second part of the experiment involved a series of rabbit corneal SHG images obtained from the temporal natural healing after alkali chemical burn [21]. All corneas used here were excised with a trephine right after death and immersed in phosphate-buffered saline solution. The specimens were not stained. Further details on tissue maintenance procedure, and the microscope operation can be found elsewhere [21,22].

### 2.3. Algorithm and Method of Analysis

The method proposed herein is divided into various steps. The first one involves the calculation of the intensity gradient of the images, taken as a two-dimensional function *I* (*i,j*) with *i* and *j* being the coordinates identifying each pixel from the image in rows and columns, respectively. This gradient is a two-dimensional vector, whose direction is along the increased intensity, and the magnitude informs of the rate of change. Mathematically, the gradient along a particular direction is the partial derivative of the function when the other directions or coordinates remain constant.

Three parameters are configured during the gradient calculation: resolution (R), threshold value (T), and binning (B). Resolution is the number of averaged pixels across the image for the gradient calculation. A resolution of *n* represents a calculation where the derivative was obtained averaging the change along *n* pixels for every direction. The threshold indicates a minimum value for the magnitude of the gradient vectors to be accounted in the analysis. Below that value, the changes in intensity can be discarded. To provide physical significance to this parameter, images are systematically normalized to 1 so that the value of the threshold can be compared among images.

On the other hand, the directions of all the vectors are represented using a positive angle *θ* ranging from 0 to 180 deg (i.e., first and second quadrants). Then, those vectors that exhibit directions from 0 to −180, or from 180 to 360 deg (i.e., third and fourth quadrants), are forced to contribute to the same direction as their positive counterpart parallel vectors. Provided that each gradient vector from the set might exhibit any direction in this range, a specific parameter named as direction binning is used to discretize the angles describing the vector direction. This can take values from 1 up to 180 deg. For instance, an angle tolerance or binning of 5 deg groups any angle *θ* described by the gradient vector within the range (0,5] deg into the same single direction, and so on, up to the set of angles within (175, 180] deg. Consequently, a binning of 5 would generate a set of 36 orientations.

Once the set of gradient vectors are computed, the normalized probability density function (*PDF*) of the directions of the vectors is generated. This *PDF* describes the normalized likelihood of a gradient vector from the image to acquire an orientation on a particular value or range provided by the parameter binning explained before.

The third and final step consists of fitting the *PDF* to the following Equation (1):

(1) $$PDF\left(\theta \right)=\sum_{k}\;a_{k}\cdot \exp\left(-\frac{{\left(\theta -b_{k}\right)}^{2}}{2\cdot c_{k}^{2}}\right)+d.$$

This *PDF* fitting model is the summation of *k* Gaussian functions (with the angle *θ* as independent variable) over a common pedestal *d*. To fit the function, a curve fitter application is employed from MATLAB (MATLAB Version R2024a, The MathWorks Inc., Natick, MA, USA). It performs a least squares parametric fitting over the custom Equation (1). The number of Gaussian functions accounts for the number of different main orientations of the fibers found in adjacent stromal layers. The parameter *a_k_* shows the height of the *k-th* Gaussian over the common pedestal *d*, *b_k_* indicates the main direction of the fibers, and *c_k_* represents the width of the corresponding Gaussian function.

**Figure 1:**
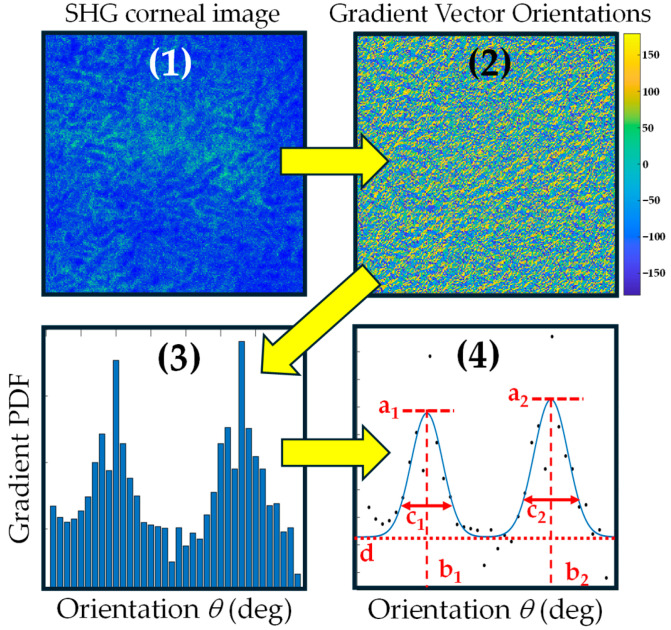
(**1**) Flowchart of the algorithm starting from a porcine corneal SHG image; (**2**) a gradient vector orientations map; (**3**) PDF of the gradient orientations; (**4**) and curve fitting to Gaussian functions and subsequent parameters.

Figure 1 presents a flowchart summarizing the algorithm, showing graphically the significance of every parameter. The starting point is the corneal SHG image (1); the algorithm first obtains the orientations of the gradient vectors in the range [−180, +180] degrees over the image, creating a color map depicted in panel (2) of the figure. The normalized *PDF* is then calculated (3) as a function of the angles, within bins of equal width (in this example, the angle bin was 5 degrees). The angles were represented from 0 to 180 degrees, as explained above. Finally, the set of Gaussian functions, in this case 2, is fitted, and the parameters *a_k_*, *b_k_*, *c_k_*, and *d* are automatically obtained (4).

## 3. Results

To test and show the capabilities of the new method of analysis, different SHG images were processed following the algorithm described above. A set of images from different species were employed to study the impact of the parameters on the performance of the algorithm and to characterize and compare the structure of collagen fibers across distinct animal models. Later in a separate subsection, the new method exhibited its potential to track and numerically characterize the changes associated with the degradation and healing process in a cornea from a rabbit model.

### 3.1. Corneal Images and Algorithm Parameters Optimization

**Figure 2:**
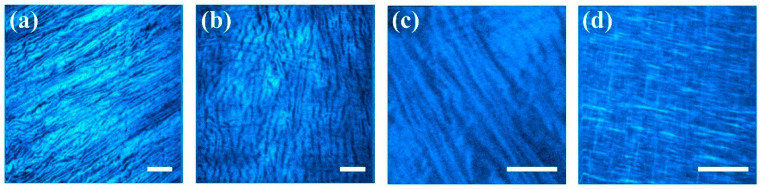
SHG images from the corneal stroma of different species: (**a**) porcine; (**b**) bovine; (**c**) human; (**d**) avian. Bar length: 50 μm. The laser power was set to 30 mW. Image resolution in pixels: (**a**,**b**), 512 × 512; (**c**,**d**), 256 × 256.

Representative SHG images from the corneal stroma of different species are displayed in Figure 2. From these, the collagen distribution was assessed as described in the previous section. Images were normalized to their maximum to span the gray scale similarly. By visual inspection it can be inferred that the quality and structure of the collagen fibers differed across samples.

Once the SHG images are acquired, the intensity gradient is computed. Its optimal calculation requires an adjustment of the three parameters explained above prior to obtaining the *PDF* with the corresponding Gaussian model fitting. These parameters are the resolution for the calculation of the intensity gradient in pixels (R), the threshold for the magnitude of the gradient vector (T), and the angle tolerance or binning (B) in the estimation of the orientation of the gradient vectors.

**Figure 3:**
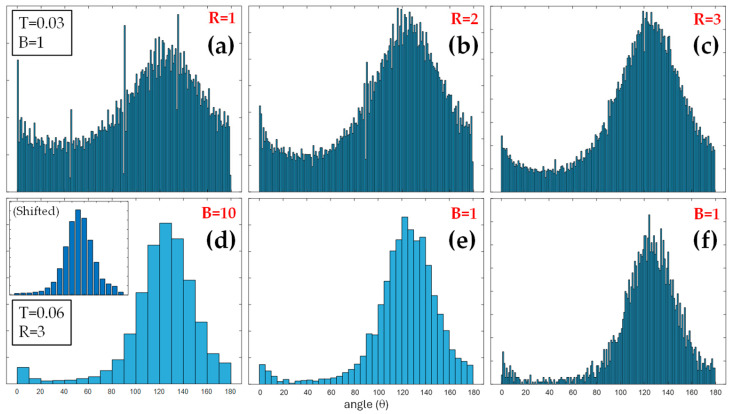
Normalized *PDF*s of intensity gradient vectors as a function of the relative orientation of the vector for different resolution (R), threshold (T), and binning (B) parameter values: (**a**) R = 1, T = 0.03, B = 1; (**b**) R = 2, T = 0.03, B = 1; (**c**) R = 3, T = 0.03, B = 1; (**d**) R = 3, T = 0.06, B = 10; (**e**) R = 3, T = 0.06, B = 1; (**f**) R = 3, T = 0.06, B = 1. These results correspond to the SHG image in Figure 2a.

To better illustrate the significance and impact of manipulating such parameters, an example is presented in Figure 3, where the SHG image in Figure 2a was used as the input for the algorithm. This presents the *PDF* histograms of the intensity gradient vectors as a function of their relative orientation for different values for the adjustment parameters. The area under the *PDF*s was systematically normalized to one so that the vertical scale was modified to enhance visualization accordingly. The independent variable *θ* (i.e., the angle) in the histograms ranged from 0 to 180 deg.

Figure 3a presents the histogram corresponding to R = 1, T = 0.03, and B = 1. This T value was assigned as 10% of the maximum magnitude of the gradient vectors found herein. Since B = 1, the discretization of the gradient vector angles exhibited 180 bars. Moreover, by visual inspection, it can be observed that four bars exhibit significantly higher values as compared to the rest of bars in their neighborhood. These correspond to angles of 0, 45, 90, and 135 deg, and they are essentially associated with intensity noise in pixels of low or no signal. In that case, when the intensity presents random values, any angle is equally probable, and the orientations are distributed across these four angles.

The value R = 1 indicates that the intensity gradient was calculated pixel to pixel, and it was the main factor responsible for the emergence of the four aforementioned bars in the histogram. Figure 3b,c depict the results when R value increases to 2 and 3, respectively, maintaining the rest of the parameters. The four noisy bars present in Figure 3a progressively decrease to reach values according to their surroundings, as shown in Figure 3c.

In the bottom row panels of Figure 3, the values were set to T = 0.06, R = 3, and B = 10, 1, and 1 in panels (d), (c), and (f), respectively. The binning increase causes a *PDF* smoothing, adopting a more Gaussian-like profile at the cost of impairing the capability to discern the orientation of the gradient vectors. This circumstance could be interesting, however, in certain cases. It must be noticed that the orientation of the vectors is provided with reference to the orientation of the image, which must be acquired with controlled positioning to provide physical significance to the angle itself. Otherwise, the angle distribution should be obtained solely as relative orientation within the frame of the image. The inset in Figure 3d shows the same histogram shifted to bring the maximum value to the center of the scale.

This practice is useful to enhance the fitting of the *PDF* to the Gaussian model. The effect of changing the T value can be evaluated by comparing Figure 3c,f, where the value T is doubled, from 0.03 to 0.06. Augmenting the T value discards every gradient vector below that value, leading to a decrease in the *PDF* pedestal, neglecting noisy vectors.

### 3.2. Comparison of Collagen Distribution Across Species

**Figure 4:**
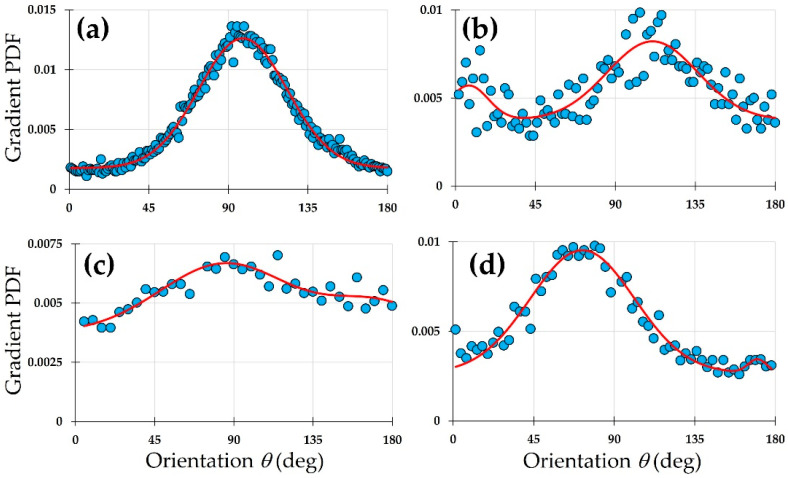
Normalized *PDF*s of intensity gradient vectors (blue dots) and curve fittings (red lines) for the SHG images of different species: (**a**) porcine; (**b**) bovine; (**c**) human; (**d**) avian.

SHG images presented in Figure 2 were analyzed with the new method proposed here. Due to the different pixel resolution of the images, both B and R were adjusted to account for it. In addition, T was set to 10% of the maximum gradient vector magnitude in every case to hold a comparable noise sensitivity across images. The results for the experimental *PDF*s and their fittings to the Gaussian model are presented in Figure 4. For better identification, these plots have been ordered, following the labels in Figure 2, so that the panels could be identified with their corneal images.

Whereas the *PDF* in panel (a) was fitted to a single Gaussian function, a double Gaussian function was used to fit the data in panels (b), (c), and (d). The use of one or two Gaussian functions was based on the statistical parameters of the fitting, specifically, the correlation coefficient. The numerical results obtained from the fitting for every image at 95% confidence are presented in Table 1 with the same labels as in previous figures.

The experimental data of Figure 4a were fitted to a single Gaussian function, exhibiting a near perfect (*R*^2^ = 0.99) correlation. This indicates the excellent fidelity of the mathematical model. This fit indicates that there is a single dominant direction for the alignment of the collagen fibers. Other orientations present a symmetrical distribution around the dominant angle, and these are associated to the width of the Gaussian (parameter *c*_1_ = 25 deg in the model, Table 1). The pedestal of the fitting (parameter *d*) was very low (0.0018) compared to others and might be associated with the noise in the image.

The *PDF* of the panel in Figure 4b was fitted to a model with two Gaussian functions. It can be observed that the red curve evolves fairly in the middle of the data cloud, following the trend. This dispersion of data around the curve is responsible for the relatively lower *R*^2^ coefficient as compared to the rest of the fittings. The physical significance of such spread in the points of the plot obeyed the lack of a true alignment of elongated collagen fibers. They present a wavy shape instead. Moreover, the second dominant direction of the fibers was responsible for the secondary peak of the Gaussian models. These peaks were separated by 103.3 deg (see values *b*_1_ and *b*_2_ in Table 1). On the other hand, a close visual inspection of the bovine SHG image in Figure 2b reveals a subtle set of fibers appearing in a near-perpendicular direction to the main direction of alignment. This agrees with Figure 4b.

Despite the lower resolution of the human cornea SHG image of Figure 2c, a double Gaussian function also modelled the *PDF* well, with a high correlation *R*^2^ = 0.78 (Figure 4c). By inspecting the image, some subtle fibers perpendicular to the dominant orientation can be detected; these are the origin for the secondary direction of fibers found in the fitting.

Finally, for the avian cornea (Figure 2d and Figure 4d), the existence of an orthogonal interweaving becomes more evident. The fit to the *PDF* presents an elevated goodness (*R*^2^ = 0.93). However, the importance of the second direction was noticeably lower in terms of the density. Both orientations were 97.6 deg apart (76 and 170 deg for b_1_ and b_2_ in Table 1).

**Figure 5:**
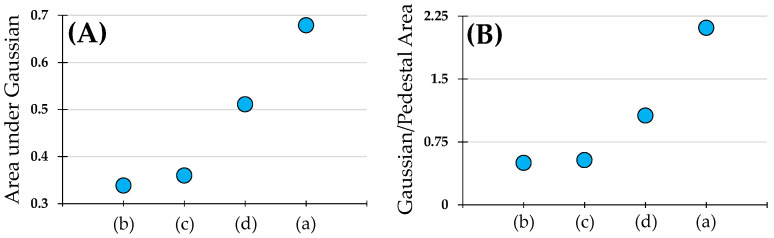
(**A**) Comparison of the Gaussian areas under the normalized *PDF* fitting function for the corneal collagen distribution across different species: (a) porcine, (b) bovine, (c) human, and (d) avian; (**B**) Gaussian/pedestal areas across the same species.

The numerical results for the fittings disclosed in Table 1 also serve as a comparison among the corneal stroma arrangements from different species. Additionally, the results on collagen distribution can be confronted with indirect parameters such as the area under the Gaussian bell or the area under the pedestal of the fitting function (Figure 5).

Figure 5A presents the areas estimated from the width and height of the Gaussian functions (parameters *a* and *c*) for each *PDF* in Figure 4. This parameter is useful when comparing fittings of single and double Gaussians. In the case of double (or more) Gaussians, the total area was calculated as the algebraic sum. Figure 5B shows the ratio between the Gaussian area and the area under the pedestal (parameter *d*). This parameter was an indicator of the noise level or, alternatively, the relative importance of zones without a defined collagen structure in the SHG images. These plots provided a simple and straightforward way to classify and quantify the order of the collagen fibers across different corneal images/species.

### 3.3. Cornea Healing in a Rabbit Model

**Figure 6:**
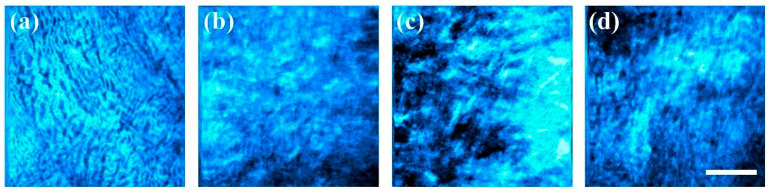
SHG images of a rabbit control cornea (**a**), and 1 (**b**), 3 (**c**), and 5 (**d**) months after chemical damage. Bar length: 50 μm. The laser power was set to 80 mW.

The new procedure was tested in a temporal series of corneal images corresponding to natural healing after a chemical burn obtained from a rabbit model [21]. SHG representative images are displayed in Figure 6. These correspond to a control sample (Figure 6a) and those acquired at different time points after the burn (Figure 6b–d). This type of temporal series is of high importance to understand the corneal wound healing process after external injury, specifically how the collagen evolves and eventually recovers.

**Figure 7:**
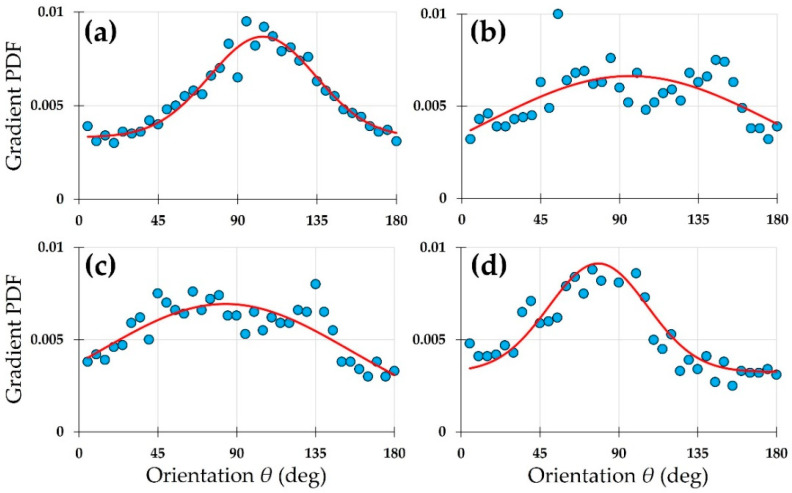
*PDF*s (blue dots) and single Gaussian model (red curves) for the SHG images of a rabbit cornea: (**a**) control; (**b**) 1 month after chemical damage; (**c**) 3 months after chemical damage; (**d**) 5 months after chemical damage.

From these images, the associated *PDF* of the intensity gradient was computed and fitted to a single Gaussian model. To better compare the results the parameters used to generate the *PDF*s were the same for all images: T = 0.05; B = 5; R = 2. The experimental *PDF*s and their associated curve fittings are displayed in Figure 7.

Since information on absolute fiber directions for each image was not provided, the relative orientation of patterns within every image did not exhibit physical significance. Then, the *PDF* data in Figure 6 were shifted so that the center of the Gaussian function (provided by parameter *b*) was around the center of the range to enhance the output of the fitting algorithm and visualization of data.

The Gaussian fit (red line) of every panel in Figure 7 followed the cloud of the *PDF* data (blue dots), although differences in the goodness of the fitting could be anticipated by simple visualization. Similarly to previous analysis in Figure 4, the squared correlation coefficient was selected to characterize the performance of the model. This statistical parameter, together with the rest of parameters obtained for the curve fitting at 95% confidence, is shown in Table 2.

**Figure 8:**
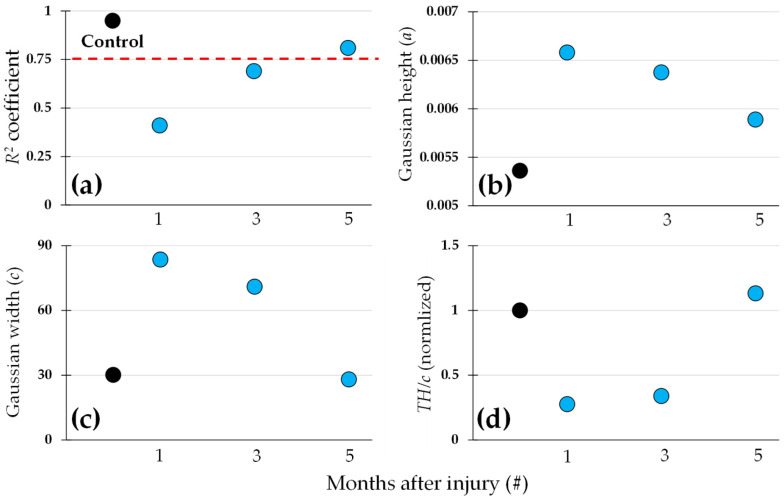
Selected parameters from the *PDF*s models from the rabbit corneas (control, 1, 3, and 5 months after chemical damage): (**a**) *R*^2^ coefficient, with a red dashed line indicating the value 0.75 in the plot; (**b**) Gaussian height; (**c**) Gaussian width; (**d**) normalized ratio of the total height of the PDF to the Gaussian width. In all the panels the black dots corresponded to the control cornea.

The coefficient *R*^2^ for the control image, before inducing the injury, is the highest one (close to 1). This indicates that the associated *PDF* to the fiber structure in a normal cornea could be modelled with a single Gaussian function. Consequently, a possible approach to characterize the corneal damage is to evaluate to what extent a Gaussian function fits well with its associated *PDF*. This is graphically presented in Figure 8a. The black dot (in all panels) corresponds to the control cornea data. The dashed red line shows the value *R*^2^ = 0.75, which could be used as a possible threshold to consider a very high goodness in the curve fitting. Whereas the values corresponding to the SHG images obtained 1 and 3 months after the injury lay below the red line, for the 5-month SHG image, this scored above.

Another possibility to quantify the evolution of the cornea is to compare the parameters of the Gaussian in the model, such the height and the width. These are depicted in Figure 8b and Figure 8c, respectively. The trend found in Figure 8a is nearly reproduced in those panels, with a clear change associated with the SHG image recorded 1 month after injury, which progressively evolved towards values close to those obtained in the control image as the time passed by.

It might be interesting also to incorporate in the analysis the pedestal of the model, which is associated with the relative strength of the noise or parts of the image with no defined structure in the fibers. Figure 8d shows a combined parameter result of the sum of the total height of the model function and the pedestal and Gaussian height, divided by the width of the Gaussian model (from Figure 8c).

## 4. Discussion

As presented herein, when the fibers within the image show a single preferential orientation, the interest is centered on computing the global order of the pattern by means of a simple Gaussian function fitting. If two directions are present, the procedure employs two Gaussian functions to fit the intensity gradient *PDF*. Biologically, this suggests that the lamellae have varying preferred orientations—an architectural feature observed in the stroma of some species. A double-Gaussian model was fitted to bovine, human, and avian corneas. This aligns with the known presence of intertwined collagen structures. Such complexity likely enhances mechanical strength and directional stiffness, which may reflect species-specific adaptations to visual and structural demands. In contrast, the pig cornea could be modeled using a single Gaussian peak, suggesting a simpler alignment pattern and potentially less lamellar intertwining. These differences highlight how the presented method can be used not only to quantify structural organization but also to offer biomechanical and evolutionary insights across species.

However, in pathological or treated tissues, more complex collagen organizations appear, and this could be expanded to models with more than two functions to be fitted. Moreover, the ability to distinguish subtle shifts in fiber orientation through *PDF* modeling could provide a valuable metric for tracking corneal healing and assessing treatment efficacy. Objectively, the statistical goodness of the fitting could provide information about the necessity of using one, two, or more Gaussian functions by inspecting the least number *k* providing the maximum *R*^2^ (the square of the Pearson correlation coefficient) in Equation (1).

This parameter *R*^2^ has been presented as a straightforward and efficient way to indicate, with a single number, how well the experimental data followed the model (i.e., fitted function), being directly related to the ordering of the collagen fibers. An example was introduced in the results presented in Figure 8. The corneal natural healing produced a *PDF* that progressively becomes more Gaussian-like (fiber collagen recovery), as described with the *R*^2^. It could be summarized that the progressive increase in *R*^2^ and decrease in Gaussian width indicated the restoration to a more organized collagen, making this method a sensitive tool for tracking stromal remodeling.

It is important to note that the healing study presented in this work used a single rabbit. This limits the statistical power of biological interpretation. The purpose of this segment was to illustrate the method’s ability to track structural changes over time. The consistent trends observed in the Gaussian fitting parameters across all four time points support the sensitivity of the approach. However, larger cohort studies will be necessary to confirm these patterns and extend the results to broader clinical or translational research.

In the proposed algorithm, vector orientations were constrained to positive angles by converting negative values to their corresponding positive equivalents. This adjustment was motivated by scenarios such as fiber detection, where, for instance, a structure oriented at 45 degrees—characterized by a local increase in intensity—may yield gradient vectors oriented at 135 degrees and −45 degrees (or equivalently, 315 degrees), both of which point toward the fiber. Retaining negative angles would result in a mirrored *PDF* around 0 degrees, potentially introducing ambiguity in the orientation representation. Converting all angles to their positive parallels eliminated this redundancy and was sufficient for accurately identifying fibers in the context considered. Nonetheless, in other applications involving different structural patterns, preserving negative angles may be advantageous. The algorithm can be modified accordingly to accommodate such cases without impacting on its overall functionality.

On the other hand, the use of the *PDF* of the magnitude of the gradient vectors has not been explored in this work, but it could provide additional information on the distribution of patterns in the image, and it might be a future direction to enhance the method, particularly with other types of samples and targets.

The current version of our algorithm requires a semi-manual adjustment of the parameters, whose effect in the experimental *PDF* were described in Figure 3. It is possible, however, to automate the process by defining optimal merit functions. This is a future goal requiring a larger number of samples. Moreover, the search for optimal parameters could be accomplished by machine or automatic learning provided a sufficient large collection of images to train the method. Using Gaussian functions was very useful for exploring other interesting parameters to classify and better understand the order and structure of the samples through the calculation of the areas under the Gaussian functions. The area is proportional to the multiplication of the height *a* by the width *c*, providing a means to compare across very different images numerically. Another area of interest was the one determined by the pedestal, related to the portion of the image presenting no structure or fibers. These possibilities could be investigated with the help of generated images or phantoms of known characteristics. In the first stages of this work, artificial ground-truth data sets were employed to refine the algorithm [53].

Compared to FT and ST methods, our gradient-based approach with Gaussian *PDF* fitting offers several advantages. FT is global in nature and fails to resolve localized disorganization or multi-directional layering, particularly in corneas with regional damage or healing (e.g., keratoconus or scars). Although some authors have tried to improve the FT procedure [29], the method proposed herein is simpler and straightforward. ST analysis, while more localized, remains sensitive to low-contrast and noisy regions. As shown in Figure 4, even in relatively noisy or complex cases like the bovine and avian samples, our method maintained robust fits (R^2^ = 0.68–0.93) and captured secondary orientations, which are typically missed or misrepresented by FT and ST. Additionally, the ability to adjust gradient parameters (R, T, B) provides flexibility without compromising reliability, unlike the often rigid and parameter-sensitive nature of FT/ST.

## 5. Conclusions

In this study, a novel quantitative method for analyzing collagen fiber organization in corneal tissues has been reported. This approach is based on statistical analyses of intensity gradients and their *PDF*s. The combination of gradient vectors and Gaussian function modeling offers an objective, flexible, and scalable tool for evaluating collagen distributions in both healthy and diseased corneas.

The method has been validated with corneas of different species, which demonstrated its versatility and ability to identify specific collagen patterns presenting single or double dominant orientations. These differences were quantitatively captured through Gaussian model parameters, highlighting the method’s potential for comparative anatomical and biomedical research.

The procedure also proved sensitive to subtle changes in collagen distribution, as shown in a corneal temporal healing model. It effectively tracked the progressive restoration of fiber alignment over five months following chemical injury, showing a clear transition from disordered to fairly ordered structures. This capacity to monitor structural remodeling over time demonstrates its usefulness for studying healing, disease progression, and evaluating therapeutic interventions.

These features could be beneficial for clinical applications in keratoconus. In this condition, early-stage fiber disorganization appears subtle and localized. Our approach could provide early diagnostics or eventually be used to monitor biomechanical restoration after cross-linking therapies. Similarly, in postsurgical corneas (e.g., post-PRK or LASIK), it may offer insight into the recovery of stromal collagen.

An important advantage of this method is its robustness for imaging noise and artifacts, outperforming conventional techniques, which often fail in low signal-to-noise conditions or when fibers are locally disrupted. Moreover, the ability to adjust certain parameters (T, R, and B) ensures adaptability to various imaging conditions and tissue types without compromising quantitative consistency.

Overall, this work represents a step forward for advanced corneal imaging and analysis by providing a powerful framework for assessing collagen fiber organization with high accuracy and reproducibility. Its broad applicability ranges from basic research to clinical diagnostics, including pathological evaluations. Future work should focus on automating parameter optimization using machine learning to enable high-throughput analysis and extend its use to other collagen-rich tissues. Integrating this methodology with AI-based image recognition systems may facilitate real-time diagnostic assessments in ophthalmic clinics [54].

## Figures and Tables

**Table 1:** Parameters obtained from the fittings to the *PDF*s in Figure 4.

SHG Image	*a*_1_	*b*_1_	*c*_1_	*a*_2_	*b*_2_	*c*_2_	*d*	*R* ^2^
(a)	0.0108	98.2	25.0	0	0	0	0.0018	0.99
(b)	0.0019	7.6	10.9	0.0045	110.9	25.5	0.0038	0.68
(c)	0.0029	85.4	37.3	0.0013	171.3	27.1	0.0037	0.78
(d)	0.0069	72.2	29.1	0.0008	170.0	5.2	0.0027	0.93

**Table 2:** Parameters obtained from the fittings to the *PDF*s in Figure 6.

	*a*	*b*	*c*	*d*	*R* ^2^
Control	0.0054	104.1	30.1	0.0033	0.95
1 month	0.0066	96.1	83.6	0.0001	0.41
3 months	0.0064	83.5	71.0	0.0006	0.69
5 months	0.0059	78.2	28.0	0.0033	0.81

## Data Availability

A beta version of the MATLAB code partially used in the study can be shared upon reasonable request to the authors. Numerical data presented graphically in the figures are also available upon request.
